# Autophagy delays progression of the two most frequent human monogenetic lethal diseases: cystic fibrosis and Wilson disease

**DOI:** 10.18632/aging.101736

**Published:** 2018-12-19

**Authors:** Luigi Maiuri, Guido Kroemer

**Affiliations:** 1 Department of Health Sciences, University of Eastern Piedmont, Novara, Italy; 2 European Institute for Research in Cystic Fibrosis, San Raffaele Scientific Institute, Milan, Italy; 3 Equipe11 Labellisée Ligue Nationale contre le Cancer, Centre de Recherche des Cordeliers, Paris, France; 4 INSERM U1138, Centre de Recherche des Cordeliers, Paris, France; 5 Université Paris Descartes, Paris, France; 6 Metabolomics and Cell Biology Platforms, Institut Gustave Roussy, Villejuif, France; 7 Pôle de Biologie, Hôpital Européen Georges Pompidou, AP-HP, Paris, France; 8 Karolinska Institute, Department of Women's and Children's Health, Karolinska University Hospital, Stockholm, Sweden

**Keywords:** aging, Beclin 1, ion channels, hepatosteatosis, heavy metals

## Abstract

Cystic fibrosis (CF) and Wilson disease (WD) are two monogenetic, recessively inherited lethal pathologies that are caused by ionic disequilibria. CF results from loss-of-function mutations in CF transmembrane conductance regulator (CFTR), a channel that conducts chloride across epithelial cell membranes, while WD is due to a deficiency of ATPase copper transporting beta (ATP7B), a plasma membrane protein that pumps out copper from cells. Recent evidence suggests that both diseases are linked to perturbations in autophagy. CFTR deficiency causes an inhibition of autophagic flux, thus locking respiratory epithelial cells in a pro-inflammatory state and subverting the bactericidal function of macrophages. WD is linked to an increase in autophagy, which, however, is insufficient to mitigate the cytotoxicity of copper. Pharmacological induction of autophagy may delay disease progression, as indicated by preclinical evidence (for CF and WD) and results from clinical trials, in particular in CF patients with the most frequent *CTRT* mutation (*CFTRdel506*). Thus, CF and WD exemplify pathologies in which insufficient autophagy plays a major role in determining the chronology of disease progression, much like the pace of ‘normal’ aging that is dictated by disabled autophagy as well.

Autophagy is probably the best-established anti-aging mechanism. As organisms age, their autophagic capacity declines [1]. Measures to increase autophagy by genetic manipulation (such as overexpression of essential autophagy genes including *Atg5* or introduction of gain-of-function mutations in the gene coding for Beclin 1, *Becn1*) [2,3], caloric restriction or periodic fasting [4], provision of agents that mimic the biochemical consequence of caloric restriction (“caloric restriction mimetics”) [5–7], as well as by other pharmacological inducers of autophagy (such as rapamycin) [8,9], can extend health span and longevity [10,11]. This has been shown for many model organisms (yeast, nematodes, flies, mice) and may apply to non-human primates (in which caloric restriction has beneficial effects) [12] and humans (in thus far that a diet rich in the autophagy inducer spermidine correlates with reduced mortality) [13,14]. These antiaging effects of autophagy may be explained by its contribution to renew (and hence to rejuvenate) the cytoplasm of cells, thereby counteracting many if not most of the molecular and cellular hallmarks of aging [15]. In other words, autophagy may be conceived as (one of) the most important process(es) that antagonize(s) the time-dependent deterioration affecting macromolecules (proteins, ribonucleotides, mitochondrial DNA, membrane lipids etc.) and cytoplasmic organelles (i.e. all organelles except the nucleus), thus acting to decelerate the biological clock [16,17].

The two most frequent lethal monogenetic diseases affecting humans are cystic fibrosis (CF, also called ‘mucoviscidosis’) and Wilson disease (WD). As any other disease, both progress with age, though with rather distinct kinetics. CF kills between childhood and early adulthood, while Wilson disease kills middle-aged adults. Surprisingly, insufficient autophagy appears to be a major determinant of disease pathogenesis for both CF and WD (Figure 1).

**Figure 1:**
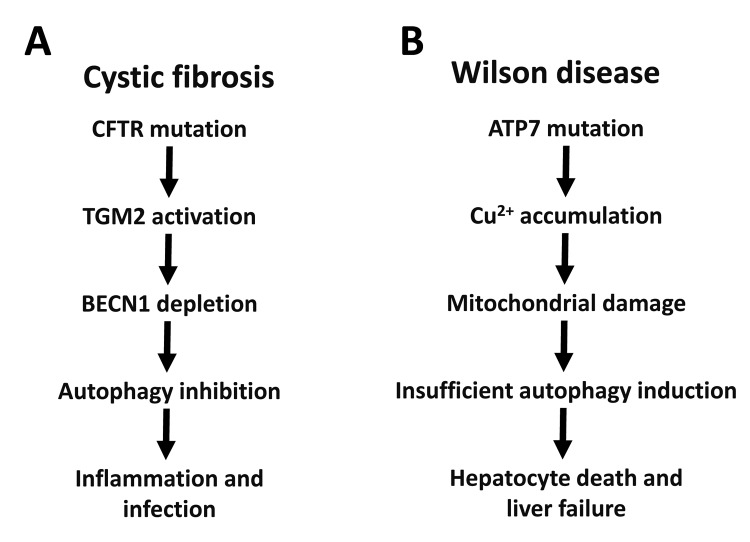
**Autophagy in cystic fibrosis and Wilson disease.** The relationship between autophagy and CFTR mutations in cystic fibrosis (**A**) or ATP7B mutations in Wilson disease (**B**) are depicted. BECN1, Beclin 1; TGM2, transglutaminase 2.

CF is due to loss-of-function mutations of the cystic fibrosis transmembrane conductance regulator (CFTR), reducing the expression or function of this chloride channel at the plasma membrane. Among 2000 different *CFTR* mutations, the most frequent one is *CFTRdel506*, accounting for the pathogenesis of 70-90% of all CF cases [18]. As a consequence of CFTR mutations, the function of epithelia (mostly in the lung but also in the gastrointestinal tract) and macrophages are compromised, ultimately causing defective clearance of mucus and infectious microorganisms [19,20]. This then triggers chronic pulmonary infection and inflammation as the primary cause of morbidity and mortality. The treatment of cystic fibrosis remains largely symptomatic, although drugs that increase the expression or improve the function of CFTR are being developed. Importantly, the CFTR defect (be it a lack of expression or a lack of function) compromises autophagy by several mechanisms including the depletion of the essential pro-autophagic protein Beclin 1 (BECN1), locking the cell in a state of deficient proteostasis [18,21]. Successful pharmacological treatment of patients bearing the *CFTRdel506* mutation with a combination of epigallocatechin gallate (EGCG, an inhibitor of the autophagy-inhibitory acetyl transferase EP300, an important regulator of autophagy) [22–25] and cysteamine (an inhibitor of transglutaminase-2) can induce autophagy in vivo, and enhance the expression of the mutated CFTRdel506 protein at the cell surface, of nasal respiratory epithelial cells from CF patients [26–28]. Of note, mice that bear the *CFTRdel506* mutation respond to this combination therapy (EGCG plus cysteamine) only if they are autophagy-competent, yet fail to do so, if they are autophagy-deficient due to the heterozygous knockout of *Becn1*. These experiments confirm that autophagy is required for the treatment to work [18,21].

WD results from the loss-of-function mutation of the gene coding for ATPase copper transporting beta (*ATP7B*), a plasma member protein that pumps out copper from cells [29]. Depending on the nutritional copper uptake and modulatory factors (such as obesity, which accelerates disease pathogenesis), copper then accumulates in particular cell types (mostly in hepatocytes, but also in cardiomyocytes and neurons) beyond a critical threshold that causes cell death, hepatic inflammation and insufficiency (and more rarely cardiomyopathy and neurodegeneration). Excessive cytosolic copper electrophoretically enriches in mitochondria, causing the crosslinking of proteins from the inner and the outer membrane of these organelles, ultimately resulting in mitochondrial destruction and cell death [30]. The only known treatments of WD aim at reducing the copper content in the diet and at chelating copper by suitable molecules such as penicillamine [31]. Recent evidence suggests that WD pathogenesis is also linked to autophagy. Indeed, the livers of WD patients and those of *ATP7B*^-/-^ rats manifest an increase in autophagic flux [32]. In vitro experiments demonstrate that excessive incorporation of copper into cells triggers autophagy, which acts a cellular defense mechanism to reduce the probability of cell death. Hence, autophagy has a cytoprotective function that is, however, insufficient to avoid the pathogenesis of WD [32]. That said, it remains to be determined whether pharmacological stimulation of autophagy would reduce copper toxicity in WD patients.

The aforementioned results suggest that autophagy plays a prominent disease-decelerating function in both CF and WD (Figure 1). Of note, these finding may have broader implications. Indeed, recent evidence suggests that CFTR function of enterocytes is inhibited in celiac disease (also called ‘gluten enteropathy’), due to its direct inhibition by gluten-derived peptides [33]. As in CF, CFTR inhibition results in reduced expression of Beclin 1 protein, thus compromising autophagy. Of note, potentiation of CFTR function by suitable drugs (‘CFTR potentiators’) can reverse the pro-inflammatory effect of gluten-derived peptides and restore Beclin 1 expression [33]. Decreased CFTR expression has also been observed in several mouse models of autoimmune disease that respond to pharmacological treatment with CFTR potentiators, suggesting that defective CFTR function (and presumably its downstream consequence, autophagy inhibition) might play a rather general role in the pathophysiology of distinct disease entities [34,35]. Similarly, the accumulation of toxic heavy metals (such as cadmium, copper, lead and mercury) may play a general role in accelerating age-related diseases [36–38]. Circumstantial evidence suggests that the toxicity of heavy metals such as cadmium is counteracted by autophagy as well [39].

Based on the aforementioned examples, it is tempting to speculate that autophagy has a general role in slowing down time-dependent processes that ultimately lead to age-related diseases. Indeed, genetic defects in different autophagy-relevant genes cause a broad range of distinct pathologies across a wide spectrum of cardiovascular, infectious, inflammatory, metabolic, neoplastic, neurodegenerative diseases (Levine and Kroemer, in press). It remains to be determined, however, which would be the optimal strategy to increase autophagy for extending the health span in the general population without such gene defects.
